# A 2.4 GHz High-Efficiency Rectifier Circuit for Ambient Low Electromagnetic Power Harvesting

**DOI:** 10.3390/s24216854

**Published:** 2024-10-25

**Authors:** Jinxin Du, Ruimeng Wang, Pingyi Zheng

**Affiliations:** Sino-European School of Technology, Shanghai University, Shanghai 200444, China; wrm981223@163.com (R.W.); 24723506@shu.edu.cn (P.Z.)

**Keywords:** ambient electromagnetic energy harvesting, low power density, rectifier circuit, voltage booster circuit, coupled transmission line, conversion efficiency

## Abstract

A novel 2.4 GHz high-efficiency rectifier circuit suitable for working under very-low-input electromagnetic (EM) power conditions (−20 to −10 dBm) is proposed for typical indoor power harvesting. The circuit features a SMS7630 Schottky diode in a series with a voltage booster circuit at the front end and a direct-current (DC)-pass filter at the back end. The voltage booster circuit consists of an asymmetric coupled transmission line (CTL) and a high-impedance microstrip line (of 100 Ω instead of 50 Ω) to significantly increase the potential at the diode’s input, thereby enabling the diode to operate effectively even in very-low-power environments. The experimental measurements show that the microwave direct-current (MW-DC) conversion efficiency of the rectifier circuit reaches 31.1% at a −20 dBm input power and 62.4% at a −10 dBm input power, representing a 7.4% improvement compared to that of the state of the art. Furthermore, the rectifier circuit successfully shifts the input power level corresponding to the peak rectification efficiency from 0 dBm down to −10 dBm. This design is a promising candidate for powering low-energy wireless sensors in typical indoor environments (e.g., the home or office) with low EM energy density.

## 1. Introduction

Ambient energy harvesting (AEH) aims to capture and convert energy from the surrounding environment into usable electrical energy and is particularly useful for powering low-energy devices, such as wireless sensor networks (WSNs). By employing AEH techniques, WSNs can operate continuously and sustainably, reducing the need for external power supplies and enhancing their autonomy. Various sources of ambient energy—including mechanical vibrations, sunlight, geothermal heat, and sound waves—can be harnessed to provide a continuous power supply for WSNs [1,2]. Recently, with the rapid expansion of wireless communication and the proliferation of wireless devices, electromagnetic (EM) energy has emerged as a particularly promising source for AEH [3,4,5]. For instance, cellular and RFID systems typically transmit around 10 watts (W), while mobile devices and Wi-Fi systems transmit around 100 milliwatts (mW) [6]. However, EM power density diminishes sharply with the transmission distance, resulting in very low power levels (mWs or µWs) at the receiver, which in turn leads to low harvesting efficiency and limited direct current (DC) output. Concurrently, the rapid development of ultra-low-power WSNs, particularly those equipped with energy management mechanisms, underscores the urgent need for effective energy harvesting from consistent and sustainable EM sources [7,8].

EM energy is most abundant in the 2.4 GHz band of the industrial, scientific, and medical (ISM) spectrum, commonly used by devices such as Bluetooth, Wi-Fi, and many other consumer electronics. Significant progress has been made in the development of high-efficiency rectifier circuits in this frequency band. For example, Zhao et al. [9] proposed a harmonic suppression structure that combines a series λ/12 short terminal and a parallel λ/8 open-circuit transmission line, eliminating the need for low-pass-filter (LPF) matching networks and thereby reducing insertion losses. In their work, when the input microwave power was set to 31 dBm and the DC load to 115 Ω, the microwave direct-current (MW-DC) conversion efficiency reached 80%. Wang et al. [10] designed a gallium arsenide Schottky diode with extremely low series resistance and high breakdown voltage for rectification. This rectifier achieved a conversion efficiency of 91% with 37 dBm of input power. These studies primarily focused on conditions with very high input power.

In typical indoor environments, however, Wi-Fi routers or hot-spots serve as the most common EM energy source and provide very limited power radiation. For instance, in a typical indoor scenario with the following components, a Wi-Fi-enhanced signal generator generating 100 mW of microwave power, a transmitting antenna with a typical gain of 5 dBi, a receiving antenna with a typical gain of 7 dBi, and a propagation distance of 1 to 5 m, then the received power at the receiving end can be as low as −22 to −8.3 dBm. Several studies have focused on rectifier circuits for relatively low input power conditions [11,12,13,14,15,16]. The HSMS 2850/2860 and SMS7630 Schottky diodes are the most frequently adopted due to their low forward voltage. Their MW-DC conversion efficiency ranges from 5% to 18% at −20 dBm of input power, and from 20% to 45% at −10 dBm of input power. Zhao et al. [9] proposed a rectifier circuit with two cascade coupled transmission lines (CTLs) optimized for −5 dBm of input power, achieving a rectification efficiency of 41% at −10 dBm and 58.5% at −5 dBm of input power. Similarly, Adami et al. [17] proposed a 2.45 GHz power harvesting wristband where the rectifier circuit was designed for −20 dBm of input power, with rectification efficiencies of 33.6% at −20 dBm and 55% at −10 dBm of input power. In addition, a dual-band rectifier circuit [18] and a broadband [19] rectifier circuit based on CTLs were investigated, achieving efficiencies of 25% at −10 dBm and 45% at 0 dBm of input power. Some studies have also attempted to explore power harvesting under even lower input power conditions. For example, the rectenna designed by Olgun [11] could harvest enough energy from a commercial RFID reader to power a 1.6 V LED, with a conversion efficiency of 8% at 25 dBm of input power. The wristband proposed by Adami [17] was able to generate a net DC output at an input power of −24.3 dBm. In summary, although these rectifier circuit designs can work properly under sub- −10 dBm input power conditions, their efficiency remains unsatisfactory. Most achieve their maximum rectification efficiency at input power levels much higher than 0 dBm, and there are still very few rectifier circuits specifically designed for efficient operation at sub- −10 dBm input power levels.

In this paper, we present a novel, high-efficiency rectifier circuit that operates at low input power levels (−20 to −10 dBm) in the 2.4 GHz band. The circuit incorporates a low forward voltage SMS7630 Schottky diode, a voltage booster circuit, and a DC-pass filter. The voltage booster circuit was built with an asymmetric CTL and a high-impedance microstrip line to boost the potential of the diode’s input. Compared to existing designs, the proposed rectifier circuit reaches a comparable MW-DC conversion efficiency of 31.1% at −20 dBm of input power, and a much higher conversion efficiency of 62.4% at −10 dBm of input power, representing a 7.4% improvement over prior designs. This rectifier circuit can be employed to power low-energy wireless sensors in typical home or office environments.

The remainder of this paper is organized as follows: Section 2 details the principle, design, and optimization process of the proposed high-efficiency rectifier circuit; Section 3 presents the measured performance of the rectifier circuit, and the comparison to the most relevant reported designs; Section 4 outlines the analysis and discussion; and finally, Section 5 provides a general conclusion about this contribution and outlines directions for future research.

## 2. Materials and Methods

Typically, a rectifier circuit consists of a rectifier diode which converts guided microwaves into DC power, a DC-pass filter which allows only DC power to flow to the load, an impedance matching network that facilitates the transfer of the microwave captured by the receiving antenna to the diode, and a DC load to receive the output DC power. The MW-DC conversion efficiency, or rectification efficiency, is a crucial performance metric for a rectifier circuit. It represents the ratio of the output DC power to the input microwave power. The conversion efficiency is tightly related to several factors, including the operating frequency, the input EM power, the diode type, the rectifier circuit structure, the impedance matching network, and the DC load resistance.

**Figure 1:**
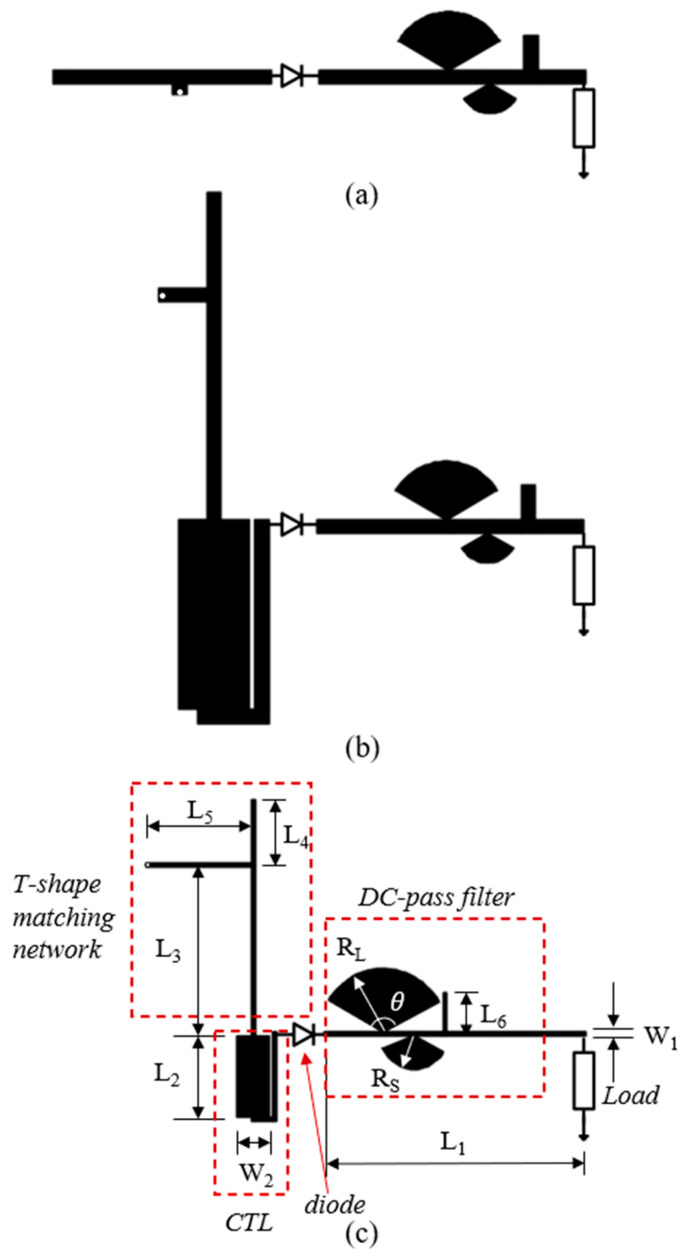
Design process of the rectifier circuit: (**a**) basic design with a 50 Ω microstrip line; (**b**) intermediate design with a CTL and a 50 Ω microstrip line; (**c**) final design with a CTL and a 100 Ω microstrip line.

Rectifier diodes are well-recognized as the most critical components in a rectifier circuit. Choosing the appropriate diode requires the careful consideration of the specific application. In this work, we adopted the SMS7630 Schottky diode (Skyworks Solutions, Inc., Irvine, CA, USA) in our design due to its ultralow forward voltage (60 to 120 mV at 0.1 mA [20]), making it ideal for low-input-power conditions. The rectifier circuit was printed on a F4B substrate with a thickness of 1 mm, a relative dielectric constant of 2.2, and a loss tangent of 0.001. The design and optimization process of the rectifier circuit are illustrated in Figure 1.

### 2.1. Basic Design of the Rectifier Circuit

**Figure 2:**
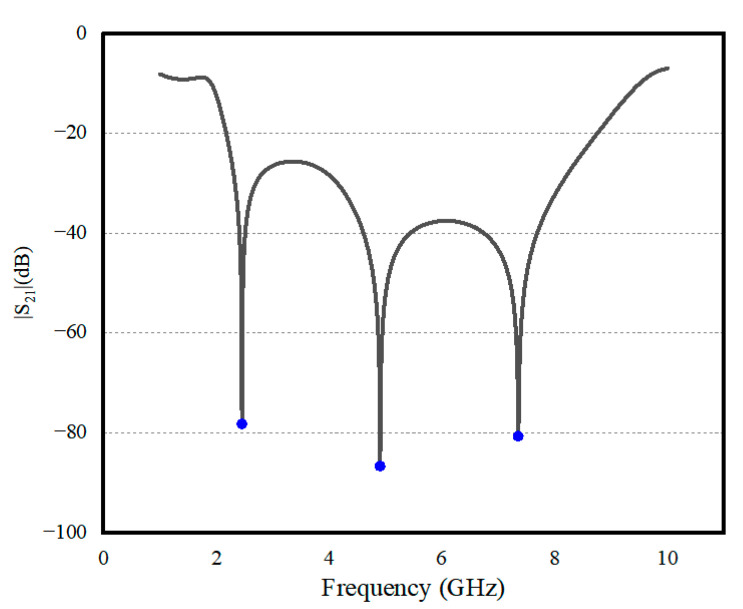
Transmission coefficient of the DC-pass filter.

Figure 1a shows the basic structure of the rectifier circuit, which is the starting point of the design process. The rectifier diode is connected in series with a 50 Ω T-shape short-circuit impedance matching network at the front end and a DC-pass filter at the back end. The T-shape matching network was optimized by using the Smith Chart method under the condition of −20 dBm of input power. The filter consists of two sector structures and an open stub line for harmonic blockage. The large and the small sectors are responsible for blocking the fundamental and the second-order harmonic, respectively, and the open stub line is responsible for the third-order harmonic. The dimensions of the filter were optimized using a parametric study approach, employing the “*Optimization*” feature of the software advanced design system (ADS 2022, Keysight Technologies, Inc., Santa Rosa, CA, USA). As shown in Figure 2, the transmission coefficients of the filter at these three frequencies are all very low, less than −80 dB, demonstrating effective harmonic suppression. The DC-pass filter enhances the stability of the DC voltage output and also reflects the harmonic signals into the rectifier circuit for multiple rectifications, thereby improving the MW-DC conversion efficiency to a certain extent.

### 2.2. Voltage Booster Circuit with CTL and High-Impedance Microstrip Line

The diode is a typical nonlinear electronic component, and its rectification efficiency increases with higher input power. Under low input power conditions, increasing the potential at the diode’s input (i.e., the voltage across the diode) enhances its performance. To achieve this, we propose a novel voltage booster circuit. First, an asymmetric CTL structure is added before the diode to act as a passive booster, as shown in Figure 1b. Second, a microstrip line with a high characteristic impedance of 100 Ω replaces the conventional 50 Ω microstrip line, as shown in Figure 1c. This replacement has two advantages: (1) it increases the potential of the guided electromagnetic wave (for a fixed power, higher impedance results in higher voltage) to some extent; (2) it allows the 100 Ω microstrip line to directly bridge the rectifier circuit to the receiving antenna, which also has a characteristic impedance of 100 Ω (which is easy to achieve). This avoids the need for the traditional 50 Ω impedance matching network, thereby minimizing the insertion loss. It should be noted that when transitioning from a 50 Ω to 100 Ω microstrip line, the CTL structure and the DC-pass filter must be redesigned and optimized accordingly.

**Figure 3:**
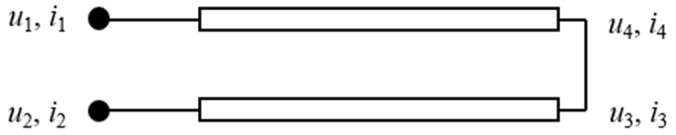
Two-port model of CTL [17].

**Figure 4:**
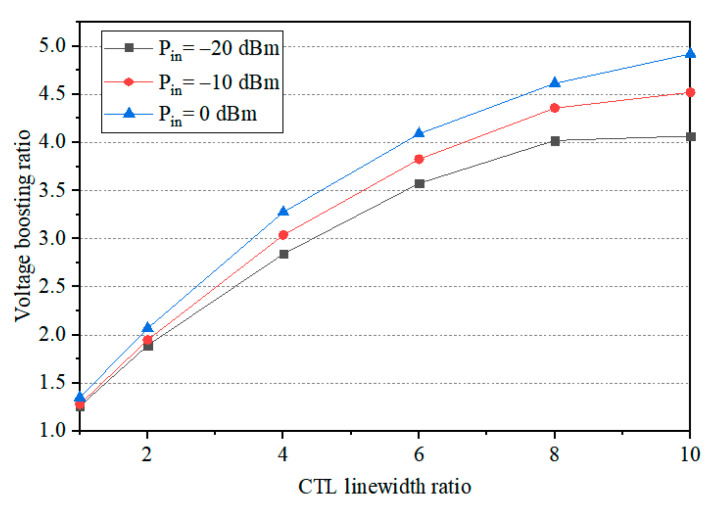
CTL voltage boosting effect as a function of the CTL linewidth ratio.

**Figure 5:**
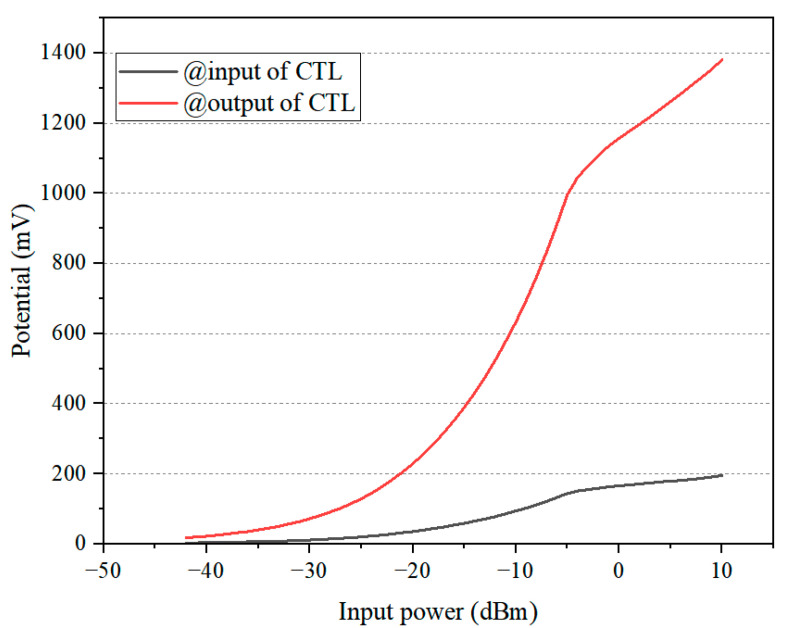
Potential at the input and output ports of the CTL as a function of the input power for a CTL linewidth ratio of 8.

An asymmetric CTL can achieve passive voltage boosting. A CTL with a short-circuited terminal can be modeled as a two-port network, as illustrated in Figure 3. The voltage boosting ratio, defined as the ratio of the output potential *u*_2_ to the input potential *u*_1_, can be estimated using the following formula [17]:

(1) $$\frac{u_{2}}{u_{1}}=\left|\frac{1}{A+\frac{B}{Z_{L}}}\right|=\left|\frac{\left[2jZ_{0e}Z_{0o}\tan\theta -Z_{L}(Z_{0e}-Z_{0o}\tan^{2}\theta )\right]}{2jZ_{L}\tan\theta +Z_{0e}Z_{0o}}\right|$$

with

$$A=\frac{Z_{0e}-Z_{0o}\tan^{2}\theta }{Z_{0e}+Z_{0o}\tan^{2}\theta },\;B=\frac{2Z_{0e}Z_{0o}j\tan\theta }{Z_{0e}+Z_{0o}\tan^{2}\theta }$$

where *Z_L_* = *u*_2_/*i*_2_, *Z*_0*o*_, and *Z*_0*e*_ are the odd- and even-mode characteristic impedances of the coupled line, respectively, and *θ* is the electrical length of the coupled line. In an asymmetric CTL where the odd-mode impedance (*Z*_0*o*_) exceeds the even-mode impedance (*Z*_0*e*_), the output potential consistently exceeds the input potential. This implies that as the guided electromagnetic wave propagates along the asymmetric CTL, its potential amplitude experiences an obvious increase. Figure 4 shows the evolution of the voltage boosting ratio as a function of the CTL linewidth ratio for the input power levels of −20 dBm, −10 dBm, and 0 dBm, respectively (results obtained via simulation in ADS). It can be observed that as the CTL linewidth ratio increases, the voltage boosting ratio also increases. However, this enhancement effect saturates as the linewidth ratio becomes larger. In our final circuit design, the CTL linewidth ratio was optimized to 8 by using a parametric study approach, employing the ADS “*Tuning analysis*” feature, since a higher linewidth ratio would not provide further boosting gain and would instead introduce processing difficulties and inaccuracies due to excessively thin or wide lines. Figure 5 illustrates the potential at the CTL’s input and output ports for a linewidth ratio of 8, showing a net voltage increase of 136 to 403 mV in the input power range of −20 dBm to −10 dBm.

**Figure 6:**
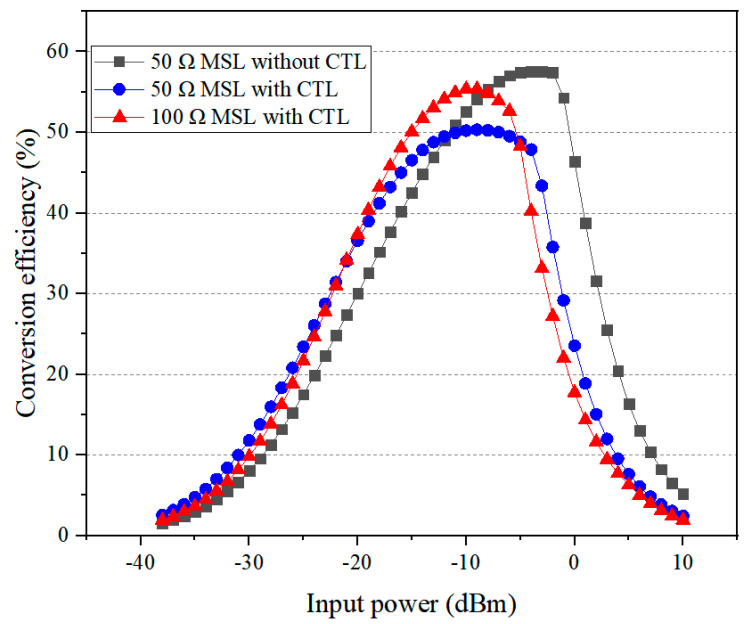
MW−DC conversion efficiency of three rectifier circuits (simulated in ADS).

Finally, the T-shape matching network, the voltage booster circuit, and the DC-pass filter were jointly optimized using the ADS “*Tuning analysis*” tool. Figure 1c illustrates the final rectifier circuit design, with dimensions: L_1_ = 54.9, L_2_ = 17.01, L_3_ = 35.25, L_4_ = 13.26, L_5_ = 22.2, L_6_ = 8.07, W_1_ = 0.86, W_2_ = 6.88, R_L_ = 13.33, and R_S_ = 7.14, all in millimeters, and *θ* = 120°. Figure 6 shows the simulation results (obtained in ADS) of the rectification efficiency as a function of the input power for three rectifier circuits: the basic design with a 50 Ω microstrip line and no asymmetric CTL structure; the intermediate design with a 50 Ω microstrip line and an asymmetric CTL structure; and the final design with a 100 Ω microstrip line and an asymmetric CTL structure. As expected, all three designs confirmed that as the input power increases, the diode operates more efficiently, thereby gradually enhancing the rectification efficiency up to a point where excessive input power causes the diode to fail. The introduction of the CTL-based voltage booster shifts the optimum input power, corresponding to the peak rectification efficiency, from 0 dBm down to −10 dBm, with some sacrifice in peak efficiency. The drawback was mitigated by replacing the 50 Ω microstrip line with a 100 Ω line. In summary, the combination of the CTL structure and the high-impedance microstrip line effectively enables operation under lower input power range without compromising the peak rectification efficiency.

## 3. Results

To verify the rectification performance of the proposed rectifier circuit, we designed a conventional planar circular monopole antenna with a characteristic impedance of 100 Ω and connected it directly to the rectifier circuit to form a rectenna.

**Figure 7:**
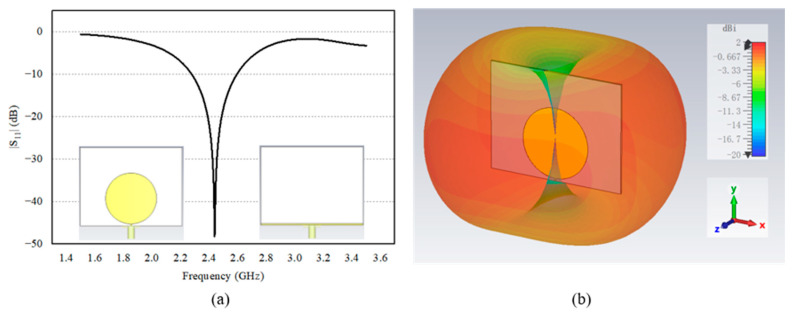
(**a**) Reflection coefficient and (**b**) radiation pattern (at 2.4 GHz) of the receiving antenna.

A circular monopole was chosen as the receiving antenna due to its wideband characteristics, which help prevent significant performance degradation in the event of a frequency shift of the rectifier circuit. Additionally, we significantly truncated the antenna’s background to reduce the overall size of the rectenna while maintaining sufficient bandwidth. Figure 7 shows the reflection coefficient |S_11_| and the radiation pattern (at 2.4 GHz) of the monopole antenna simulated in CST with a port impedance of 100 Ω. The antenna exhibits a frequency band for |S_11_|≤ −10 dB from 2.3 to 2.6 GHz and an omnidirectional radiation pattern in the H-plane.

**Figure 8:**
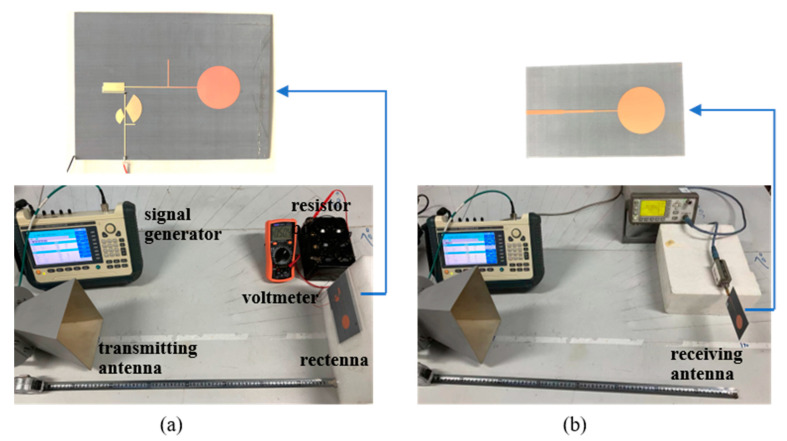
Experiment setup: (**a**) for measuring the DC output *V_out_*; (**b**) for measuring the microwave power *P_in_* captured by the rectenna.

The rectification efficiency of the rectifier circuit can be obtained from experimental measurements. Figure 8a illustrates the experiment setup for measuring the DC output. At the transmitting end, the microwave signal is generated by an AV1431 microwave signal generator and is emitted through a standard horn antenna with an aperture diagonal length of 20 cm. At the receiving end, the rectenna is mounted on a foam board, with its output connected to a variable resistor box. A voltmeter is connected in parallel with the resistor box to measure the DC voltage output. The centers of the transmitting and receiving antennas are precisely aligned, maintaining a 65 cm distance, which satisfies the far-field condition. Figure 8b shows the setup for measuring the captured microwave power by the rectenna. The same circular monopole antenna used in the previous setup is connected to an Agilent E4416A power meter, which measures the microwave power output. Note that a λ/4 transmission line was used to transfer the characteristic impedance of the antenna from 100 Ω to 50 Ω to facilitate a connection to the power meter.

The rectification efficiency of the rectifier circuit can be calculated by the following:

(2) $$\eta =\frac{P_{out}}{P_{in}}=\frac{V_{out}^{2}}{R_{Load}P_{in}}$$

where *P_out_* is the DC power received by the load, *P_in_* is the microwave power received by the rectenna, *V_out_* is the voltage across the load (measured by the voltmeter), and *R_Load_* is the resistance of the load. The input power *P_in_* is measured using the power meter.

**Figure 9:**
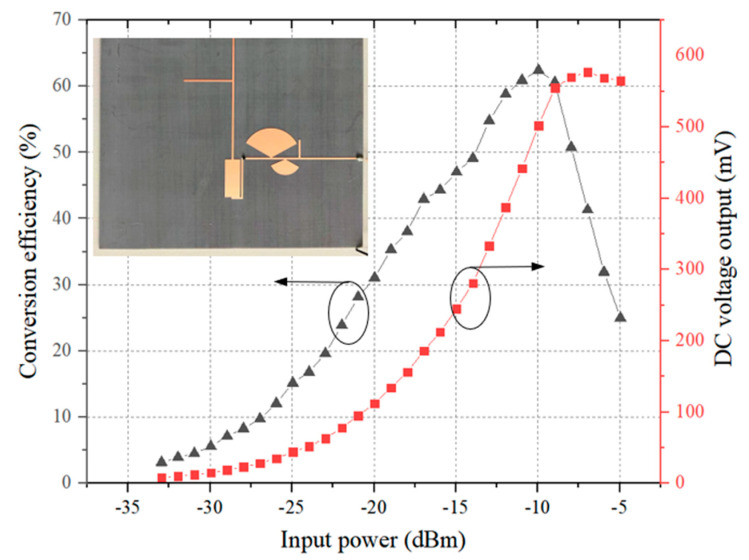
MW−DC conversion efficiency and DC voltage output of the final rectifier circuit.

Figure 9 shows the evolution of the MW-DC conversion efficiency and the DC voltage output as a function of the input power *P_in_*, with the DC load optimized to 4000 Ω. It can be observed that the rectifier circuit begins generating the net DC output at an input power as low as −30 dBm. At −20 dBm, the rectification efficiency reaches 31.1% with a corresponding DC voltage output of 112 mV. When the input power is increased to −10 dBm, the rectification efficiency rises to 62.4%, and the DC voltage output reaches 502 mV.

**Figure 10:**
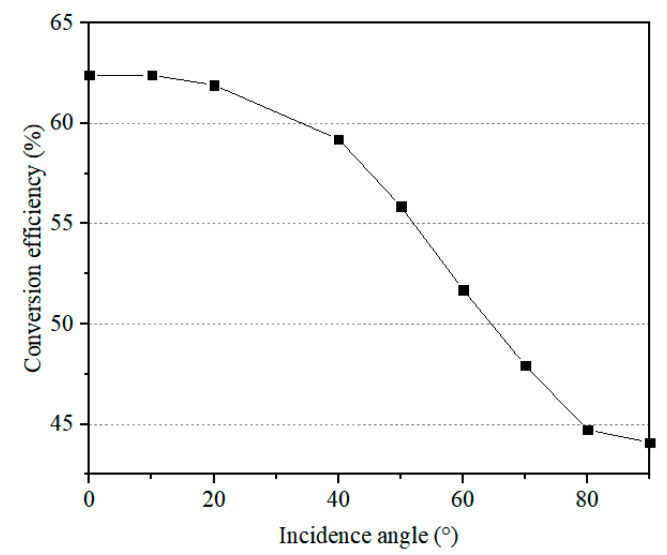
Evolution of the MW-DC conversion efficiency of the final rectifier circuit as a function of different incidence angles of microwaves.

Figure 10 illustrates how the rectification efficiency varies with different oblique incidence angles of the microwaves. It can be observed that the rectifier circuit maintains stable high efficiency for moderate oblique incidence angles of less than 25°, decreasing to 44% at an incidence angle of 90°. This trend can be attributed to the omnidirectional characteristics of the receiving antenna, as demonstrated in Figure 7b. It is important to note that the proposed high-efficiency rectifier circuit can be combined with different types of receiving antennas depending on the use cases. An omnidirectional antenna is preferred for sensors mounted on moving objects, such as the human body, while directional antennas with high gains are more suitable for sensors mounted on static objects, such as walls or furniture.

## 4. Discussion

Table 1 provides a comprehensive comparison of the proposed rectifier circuit with the state-of-the-art studies. Most existing studies aim to maximize the rectification efficiency without limiting input power. As a result, their rectification efficiency is far from being optimized under low input power conditions. Compared to the best work at low input power [17], our proposed rectifier circuit demonstrates a comparable rectification efficiency at −20 dBm of input power (31.1% vs. 33.6%) and a much higher efficiency at −10 dBm of input power (62.4% vs. 55%). Moreover, our design shifts the input power level corresponding to the peak rectification efficiency from 0 dBm down to −10 dBm, making it particularly suitable for realistic typical indoor environments.

The specially designed CTL structure plays a crucial role in achieving voltage boosting under low input power conditions, significantly enhancing the diode’s performance in such scenarios. The high-impedance microstrip line has a simple structure but can effectively enhance the voltage boosting effect—an area not thoroughly explored in existing literature. It is worth noting that the 100 Ω microstrip line may not represent the optimal impedance value for maximizing the rectification efficiency. We chose the 100 Ω microstrip line primarily to demonstrate the feasibility of voltage boosting with a high-impedance transmission line. Future research should focus on identifying the optimal impedance value, as higher impedance typically requires thinner linewidths, presenting an additional challenge. Investigating energy management mechanisms is also essential to address stability and compatibility issues to meet the requirements of various sensors. Another promising direction for future research involves developing flexible or textile-based high-efficiency rectifier circuits, which can be seamlessly integrated into wearable devices for use in indoor hospital environments.

## 5. Conclusions

Aiming at harvesting electromagnetic energy in ordinary indoor environments, this paper proposed a high-efficiency 2.4 GHz rectifier circuit suitable for working under low input power conditions. By incorporating a voltage booster network with an asymmetric CTL and a high-impedance transmission line, the circuit significantly improved the diode efficiency at low input power and effectively reduced the operating power threshold. The proposed rectifier circuit exhibited good potential for wirelessly powering low-energy sensors commonly deployed in home and office settings, as well as wearable devices for healthcare monitoring.

## Figures and Tables

**Table 1:** Comparison with state-of-the-art studies.

State of the Art	Rectifier Diode	Conversion Efficiency at −20 dBm	Conversion Efficiency at −10 dBm	Peak Efficiency at Optimal Input Power
Olgun, ISET 2010 [11]	HSMS2852	15%	45%	69% at 3 dBm
DeLong, ISAP 2016 [12]	SMS7630	5%	38%	75% at 7 dBm
Naresh, APSEM 2018 [13]	HSMS2850	15%	37%	60% at 0 dBm
Bhatt, AWPL 2019 [14]	HSMS2860	-	-	63% at 12.3 dBm
Vital, TAP 2020 [15]	SMS7630	18%	35%	70% at 8 dBm
Saito, OJAP 2022 [16]	SMS7630	13%	34%	45% at 0 dBm
Zhao, TMTT 2020 [9]	HSMS2860	-	41%	68% at 3 dB
Adami, TMTT 2017 [17]	SMS7630	33.6%	55%	65% at 0 dBm
Nam, MWTL 2023 [18]	BAT15-03W	-	25%	77.85% at 12 dBm
He, TCS 2022 [19]	HSMS2860	-	-	70% at 11 dBm
*This work*	SMS7630	31.1%	62.4%	62.4% at −10 dBm

## Data Availability

No new data were created.
